# Loss of Cell Adhesion Increases Tumorigenic Potential of Polarity Deficient *Scribble* Mutant Cells

**DOI:** 10.1371/journal.pone.0158081

**Published:** 2016-06-21

**Authors:** Indrayani Waghmare, Madhuri Kango-Singh

**Affiliations:** 1 Department of Biology, University of Dayton, Dayton, Ohio, United States of America; 2 Center for Tissue Regeneration and Engineering at Dayton (TREND), University of Dayton, Dayton, Ohio, United States of America; 3 Premedical Programs, University of Dayton, Dayton, Ohio, United States of America; 4 SupraMolecular Applied Research and Technology Center (SMART), University of Dayton, Dayton, Ohio, United States of America; National Cancer Institute, UNITED STATES

## Abstract

Epithelial polarity genes are important for maintaining tissue architecture, and regulating growth. The *Drosophila* neoplastic tumor suppressor gene *scribble (scrib)* belongs to the basolateral polarity complex. Loss of *scrib* results in disruption of its growth regulatory functions, and downregulation or mislocalization of Scrib is correlated to tumor growth. Somatic *scribble* mutant cells (*scrib*^-^) surrounded by wild-type cells undergo apoptosis, which can be prevented by introduction of secondary mutations that provide a growth advantage. Using genetic tools in *Drosophila*, we analyzed the phenotypic effects of loss of *scrib* in different growth promoting backgrounds. We investigated if a central mechanism that regulates cell adhesion governs the growth and invasive potential of *scrib* mutant cells. Here we show that increased proliferation, and survival abilities of *scrib*^-^ cells in different genetic backgrounds affect their differentiation, and intercellular adhesion. Further, loss of *scrib* is sufficient to cause reduced cell survival, activation of the JNK pathway and a mild reduction of cell adhesion. Our data show that for *scrib* cells to induce aggressive tumor growth characterized by loss of differentiation, cell adhesion, increased proliferation and invasion, cooperative interactions that derail signaling pathways play an essential role in the mechanisms leading to tumorigenesis. Thus, our study provides new insights on the effects of loss of *scrib* and the modification of these effects via cooperative interactions that enhance the overall tumorigenic potential of *scrib* deficient cells.

## Introduction

Epithelial cells are the major cell-type for all organs in multicellular organisms that organize into elaborate stratified sheets via formation of intercellular junctions, and have a distinct apical-basal polarity that is maintained during cell division [1, 2]. In order to achieve correct organ size, epithelial tissues need mechanisms that limit their proliferation, and protect tissues from damage caused by defective epithelial cells [3–5]. In *Drosophila*, defective epithelial cells that arise due to disruption of apical-basal polarity trigger a cell non-autonomous response in which either neighboring cells [6] or circulating hemocytes induce apoptosis in the mutant cells [7]. Epithelial functions such as signaling across the epithelial layer, dynamic interactions of cells with the underlying basement membrane and extracellular matrix (ECM) depend on highly organized epithelial architecture that is orchestrated by apical and basolateral junctional complexes [1, 2, 8]. This highly organized epithelial architecture is damaged and eventually lost in cancer, where malignant cells lose polarity and connections to the basement membrane causing cancer cells to become proliferative, motile (by undergoing EMT [epithelial-mesenchymal transition]), and invasive (by degrading ECM) [2, 9, 10]. Thus, the proliferation of cancer cells depends on the influence of cell-cell contacts and the cell-microenvironment interactions [11–13].

The apical junctional complexes are landmarks for the evolutionarily conserved Crumbs/Par and the basolateral Scrib polarity modules [1, 5, 14, 15]. The Crumbs (Crb) complex is formed by the association of Crb with Stardust (Sdt) and PALS1 (protein associated with Lin seven 1)-associated TJ protein (PATJ), that together play a critical role in establishing the apical domain [2]. The Par complex consists of three components: Atypical Protein Kinase C (aPKC), Cell Division Cycle 42 (Cdc42) and Partitioning Defective 6 (Par-6) that act at the apical cortex to position Bazooka (Baz) at the Adherens Junction (AJs) [9]. The basolateral Scribble complex comprises of Lethal giant larvae (Lgl) [16], Discs large (Dlg), and Scribble (Scrib) [17, 18] that are required for the formation of septate junctions, and mutations in Scribble complex components cause massive neoplastic overgrowth of mutant tissues in addition to defects in cell polarity and are referred to as “neoplastic tumor suppressor genes”. Further, the basolateral proteins are required for assembly of other junctional complexes, and are a great model system to study mechanisms of cell polarity and growth control [19, 20].

In addition to the junctional complexes described above, epithelial cells are connected to each other via adhesion molecules at the AJs that mediate stable cohesion between cells [21, 22]. These junctional complexes comprise of E-Cadherin (E-Cad), which forms a trans-dimer on adjacent cells through its extracellular domain and intracellularly binds with β-catenin and α-catenin in a junctional complex [21–23]. During normal development, the intercellular junctions provide the structural foundation for maintaining tissue architecture, and AJs are actively reorganized to allow tissue remodeling [5, 21]. Further, junctional dynamics plays a key role in how a cell responds to stress, or other signals [13, 24, 25]. Thus, the organization and maintenance of junctional complexes in epithelial cells reflects a homeostatic state, which is disrupted when junctions are damaged in conditions like cancer. Therefore, it is possible that mutations in polarity genes change cell adhesion to promote aggressive tumor growth.

We tested this hypothesis in *Drosophila scrib* mutant cells that are known to have different growth potential depending on the genotype of the mutant or neighboring cells. The vast range of phenotypes includes the slow growing *scrib* mutant cells to tumors formed by oncogenic cooperation between *scrib*^-^ and *Ras*^*V12*^ [26–30]. These phenotypic variations lead us to investigate the effects of loss of *scrib* alone, and genetic combinations that provide a growth advantage to *scrib* mutant cells on proliferation, differentiation, survival, cell adhesion and invasiveness. We show that the increased proliferation and survival associated with *scrib*^-^ cells in different genetic combinations co-relate with changes in cell adhesion. We further show that invasive potential of *scrib*^-^ cell can be uncoupled from the invasive tumor phenotype, which is exhibited only in the presence of certain oncogenic insults like oncogenic *Ras*^*V12*^.

## Materials and Methods

### Fly stocks and genetics

The *Drosophila* stocks used in this study are previously published and described in FlyBase. GFP positive MARCM clones [31] were generated in the eye-antennal imaginal discs by crossing *eyFLP*; *AyGAL4 UAS GFP*; *FRT82B TubGAL80* flies with (i) *FRT82B*, (ii) *FRT82B scrib*^*j7b3*^ or *thlacZ*, *FRT82B scrib*^*2*^, (iii) *FRT82B wts*^*X1*^, (iv) *UAS p35; FRT82B scrib*^*2*^, (v) *UAS Ras*^*V12*^; *FRT82B*, (vi) *UAS Ras*^*V12*^ *FRT82B scrib*^*2*^, and (vii) *thlacZ FRT82B scrib*^*2*^ *wts*^*X1*^ flies.

GFP negative *scrib* loss of function clones in *Minute* background [32] were generated by crossing *eyFLP*;; *FRT82B M(95A) UbiGFP* [33] flies with *thlacZ FRT82B scrib*^*2*^ or *FRT82B scrib*^*j7b3*^ flies. All experiments, except for generation of *FRT82Bwts*^*X1*^ MARCM clones (which was performed at room temperature), were performed at 25°C. Discs from wandering third instar larvae were used for all phenotypic analyses.

### Immunohistochemistry

Antibody staining was performed by using the following primary antibodies: mouse anti PH3 (1:200, Cell Signaling Technology), mouse anti DIAP1 (1:200, from Dr. Bruce Hay), rat anti ELAV (1:300, DSHB), mouse anti Armadillo (1:100, DSHB), mouse anti Fas2 (1:100, DSHB), rat anti E-Cad (1:100, DSHB), and mouse anti MMP1 (1:200, DSHB). The secondary antibodies used to detect primary antibodies were: Donkey Cy3 conjugated anti mouse IgG (1:200, Jackson ImmunoResearch) or Donkey Cy5 conjugated anti rat IgG (1:200, Jackson ImmunoResearch).

Immunohistochemistry was performed using standard protocol (Kango-Singh et al., 2002). Briefly, third instar larvae of appropriate genotypes were dissected in 1X PBS, fixed in 4% paraformaldehyde (PFA). The discs were incubated with appropriate primary (overnight incubation at 4°C), and secondary (2 hours at room temperature) antibodies. 1X PBST was used to permeabilize the tissue, and wash off unbound antibodies following each incubation. The processed tissue was mounted in Vectashield (Vector labs). A minimum of 15 discs were analyzed for each staining and genotype.

### Confocal imaging

Images (at 40X magnification) were captured using Olympus Fluoview 1000 Laser Scanning Confocal Microscope. The images were edited using Phostoshop (Version CS5, and CC).

## Results

### *scrib*^-^ cells proliferate ectopically in the presence of growth promoting mutations

It is well-documented that *scrib*^-^ cells in wild-type background undergo apoptosis that masks their neoplastic potential [30]. We generated somatic *scrib*^-^ clones in different genetic backgrounds where their elimination was compromised by (A) reducing fitness of neighboring cells by making them *Minute* heterozygous [32] [referred to as *scrib*^-^*/M* throughout the text], (B) blocking apoptosis due to overexpression of the pan-caspase inhibitor p35 [34] [referred to as *p35+scrib*^-^ throughout the text], (C) by introducing loss of function mutation in *warts* (*wts*^-^) [35]–a key player that mediates growth functions of *scrib* through the Hippo pathway [29] [referred to as *scrib*^-^,*wts*^-^ throughout the text], or (D) by activation of oncogenic *Ras* (*UAS Ras*^*V12*^) [36] in *scrib*^-^ clones [referred to as *Ras*^*V12*^,*scrib*^-^ throughout the text]. We specifically tested *scrib*^-^,*wts*^-^ combination as recently we and others have shown that AJC components like Crb, aPKC, Scrib and Lgl interact with the Hippo pathway to regulate growth [37–44].

**Fig 1:**
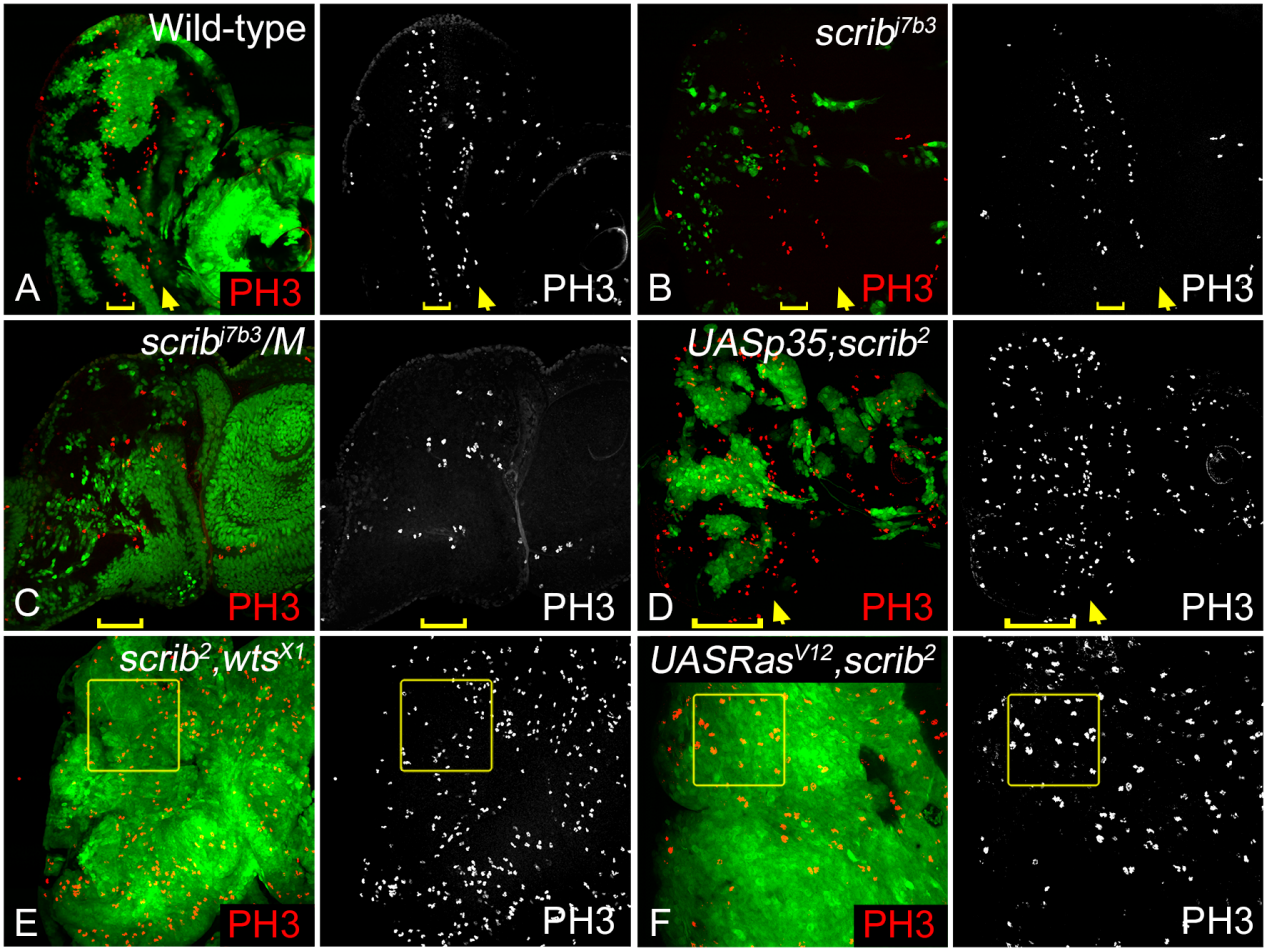
Analysis of cell proliferation in *scrib*^-^ cells with additional mutations that provide growth advantage. M-phase cells were observed in third instar eye imaginal discs by using anti PH3 antibody (red, and greyscale in A-F) to mark the proliferating cells. The panels show disc of the genotypes (A) *eyFLP; AyGAL4 UAS GFP; FRT82B TubGal80*/*FRT82B* [Wild-type], (B) *eyFLP; AyGAL4 UAS GFP; FRT82B TubGal80*/*FRT82B scrib*^*j7b3*^ [*scrib*^-^] (C) *eyFLP;+; FRT82B (M95A) ubiGFP/FRT82B scrib*^*j7b3*^ [*scrib*^-^*/M*], (D) *eyFLP; AyGAL4 UAS GFP/ UASP35; FRT82B TubGal80*/*FRT82B scrib*^*2*^ [*p35+scrib*^-^] (E) *eyFLP; AyGAL4 UAS GFP; FRT82B TubGal80*/*FRT82B wts*^*X1*^,*scrib*^*2*^ [*scrib*^-^,*wts*^-^], and (F) *eyFLP; AyGAL4 UAS GFP; FRT82B TubGal80*/*UAS Ras*^*V12*^, *FRT82B scrib*^*2*^ [*Ras*^*V12*^,*scrib*^-^]. Clones in A, B, D, E, and F are positively marked by GFP (generated by MARCM technique), and clones in C are marked by lack of GFP (generated by FLP/FRT technique). The yellow arrows (panels A-D) mark the morphogenetic furrow (MF), while the yellow brackets (panels A-D) mark the extent of the second mitotic wave (SMW). Yellow boxes in panels E and F mark ectopic PH3 within clones. All images are shown at identical magnification (40X), and orientated with posterior to the left, and anterior to the right. Genotypes, magnification, orientation and arrangement of panels mentioned in Fig 1 are consistent in Figs 2–4 and 6.

We first tested the effect of these genetic combinations on cell proliferation using an antibody against Phospho-histone H3 (PH3), which marks mitotic figures [45–47] (Fig 1). We compared PH3 profiles in eye imaginal discs containing MARCM clones that were either wild type (Fig 1A), or *scrib*^-^ (Fig 1B) with the other genetic combinations (Fig 1C–1F). Consistent with the well-documented cell cycle regulation in wild-type third instar eye imaginal discs, PH3 positive cells are seen mainly anterior to the morphogenetic furrow (MF), and in the second mitotic wave (SMW) posterior to the MF (Fig 1A) [47, 48]. This pattern remains largely unaffected in *scrib*^-^ cells in wild-type background (Fig 1B). In contrast, *scrib*^-^*/M* (Fig 1C), *p35+scrib*^-^ (Fig 1D), *scrib*^-^,*wts*^-^ (Fig 1E), and *Ras*^*V12*^,*scrib*^-^ (Fig 1F) show ectopic PH3 expression. Interestingly, of these genetic combinations only *scrib*^-^,*wts*^-^ and *Ras*^*V12*^,*scrib*^-^ clones show massive overgrowth causing a disruption in the eye imaginal disc morphology which become enlarged in case of *scrib*^-^,*wts*^-^, and show multilayered neoplastic tumor phenotypes in *Ras*^*V12*^,*scrib*^-^ double mutants. Although ectopic proliferation is seen in *wts*^*x1*^ clones (data not shown) [49], and in *Ras*^*V12*^ overexpressing clones (data not shown) [33]. These data suggest that additional mutations in *scrib* mutant cells modify their growth profile. However, the effect of these additional mutations is variable as reflected by the difference in degree of proliferation and growth. The qualitative difference in clone growth and overall eye disc size led us to ask if differentiation, survival, adhesion and invasion potential are altered in *scrib*^-^ cells in different genetic backgrounds.

### Increased survival of *scrib*^-^ cells negatively regulates differentiation

**Fig 2:**
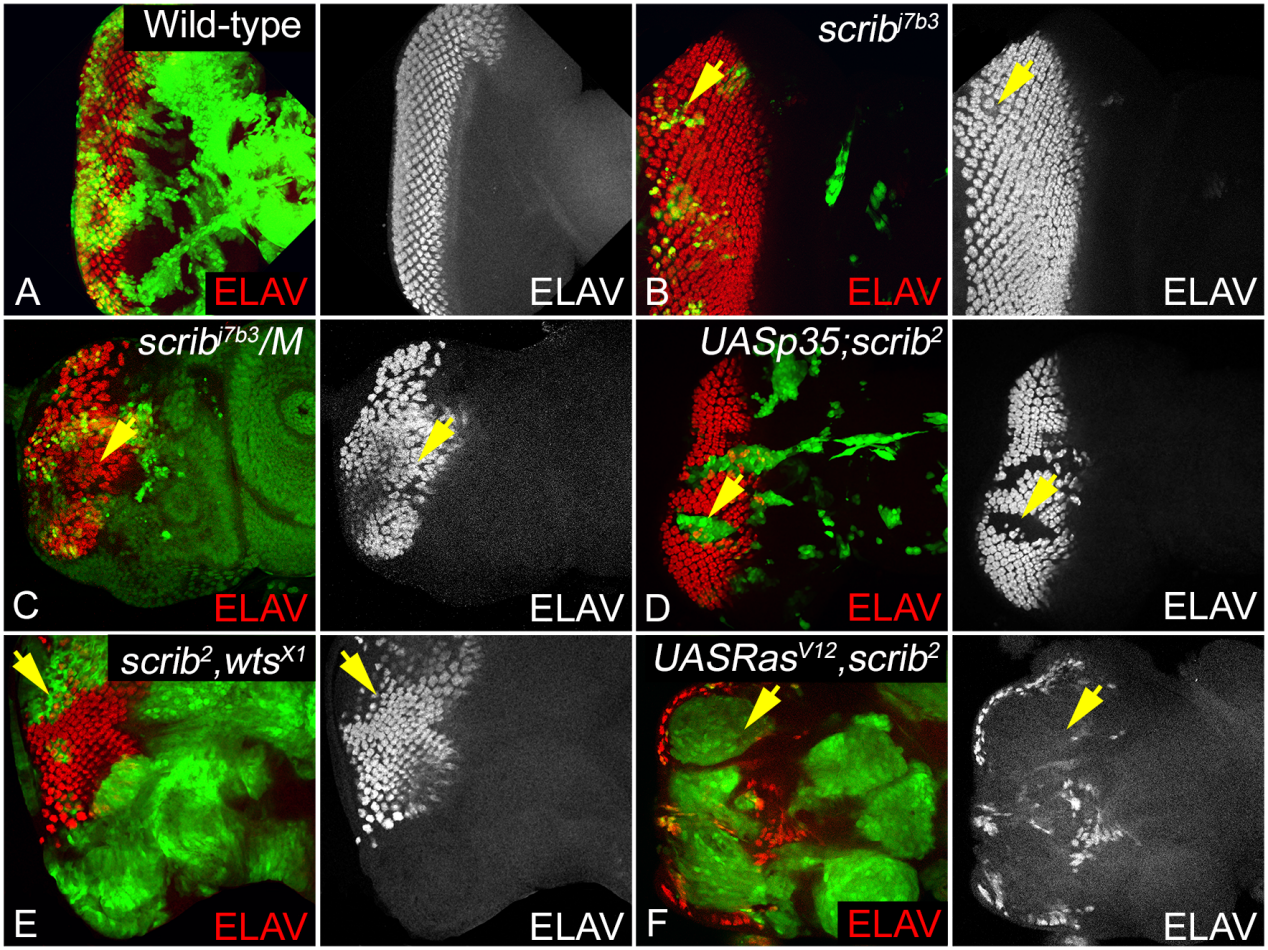
Effect on differentiation in *scrib* loss of function clones, and other genetic backgrounds. Panels show eye imaginal discs containing clones of the following genotypes (A) Wild-type (GFP-positive), (B) *scrib*^-^ (GFP-positive), (C) *scrib*^-^*/M* (GFP-negative), (D) *p35+scrib*^-^ (GFP-positive), (E) *scrib*^-^,*wts*^-^ (GFP-positive), and (F) *Ras*^*V12*^,*scrib*^-^ (GFP-positive) stained with antibody to the pan-neural marker ELAV (red, and greyscale in A-F) to assess changes in differentiation, morphogenetic furrow progression, and photoreceptor organization. Note that for all genotypes, only those clones posterior to the morphogenetic furrow are relevant for analysis. Yellow arrows in panels B-F highlight areas/mutant clones where changes in ELAV expression were assessed.

High grade tumors often have poorly differentiated cells, and altered morphology [50]. In contrast cells with poor growth potential, for example, mutations in ribosomal proteins (the *Minute* mutants) or signaling pathways, for example, loss of *Drosophila S6 kinase* (*dS6k*^-^), *yorkie* (*yki*^-^) or *Target of Rapamycin* (*dTOR*^-^*)* show poor growth but no effect on differentiation [32, 51–54]. Therefore, we checked if *scrib*^-^ cells, or the different genetic backgrounds that modify the growth potential of *scrib* mutant cells show any effects on cell differentiation. We tested for expression of Embryonic Lethal Abnormal Vision (ELAV), which is expressed in the differentiated photoreceptor neurons as a marker in the third instar eye discs (Fig 2) [55]. Compared to wild-type (Fig 2A), we did not observe any noticeable differentiation defects in *scrib*^-^ cells undergoing apoptosis (Fig 2B). However, we saw varying degree of effect on MF progression or differentiation of photoreceptor neurons when the growth potential of *scrib* mutant cells is modified in other genetic combinations (Fig 2C–2F). In *scrib*^-^*/M* discs photoreceptor neurons differentiate both in the *scrib* mutant and the neighboring *M/+* cells, however, the spacing of the ommatidial clusters and the progression of the MF are affected (Fig 2C) suggesting misregulation of furrow progression. The *scrib*^-^,*wts*^-^ double mutants show suppression of MF progression in the ventral eye margin and increased spacing between ommatidial clusters (Fig 2E), a phenotype that resembles *wts* mutant cells (S1A Fig). These phenotypes are typical of loss of Hippo pathway genes, and consistent with our earlier finding that *scrib* acts through *wts* to regulate its growth functions [29]. In comparison, *p35+scrib*^-^ mutant cells (Fig 2D) and the *Ras*^*V12*^,*scrib*^-^ mutant cells (Fig 2F) show a complete suppression of differentiation. The *Ras*^*V12*^ control clones (S1B Fig) show defects in photoreceptor organization, and regulation of furrow progression. One reason why suppression of cell death (*p35*) or overactivation of oncogenic *Ras* (*Ras*^*V12*^) cause suppression of differentiation in *scrib* mutant cells is that the signals controlling MF progression are inhibited, or alternatively changes in cell survival or cell adhesion or combinations thereof cause tumor like growth by suppressing differentiation. Therefore, we tested if these factors contribute to increased growth and tumorigenesis in *scrib* mutant cells that have increased proliferation ability.

We assessed survival potential of clones by testing expression of *Drosophila* inhibitor of apoptosis-1 protein (DIAP1), which is known to protect cells from apoptosis in several contexts including developmentally regulated apoptosis, or stress induced apoptotic response [56–62]. Compared to the ubiquitous expression of DIAP1 seen in wild-type (S2A Fig), DIAP1 levels are down regulated in *scrib*^-^ cells (S2B Fig), and *p35+scrib*^-^ (S2D Fig) clones, but induced in *scrib*^-^*/M* clones (S2C Fig). Consistent with previous reports, DIAP1 levels are robustly induced in *scrib*^-^,*wts*^-^ (S2E Fig), and *Ras*^*V12*^,*scrib*^-^ (S2F Fig) clones [28, 29]. Of note, forced suppression of apoptosis by expression of *p35* in *scrib*^-^ cells caused inhibition of differentiation despite suppression of DIAP1 suggesting that a net increase in survival of *scrib* mutant cells promotes growth and inhibits differentiation. Overall, these data suggest that upregulation of survival signaling in *scrib* mutant cells is correlates with increased growth and negative regulation of differentiation. Next, we tested if *scrib* mutant cells also induce changes in cell adhesion that enhance the overall tumorigenic potential of *scrib* mutants in the genetic backgrounds under study, or if increased survival of cells from loss of *wts* or *Ras* overexpression account for the growth phenotypes.

### Is loss of differentiation linked to changes in cell adhesion?

**Fig 3:**
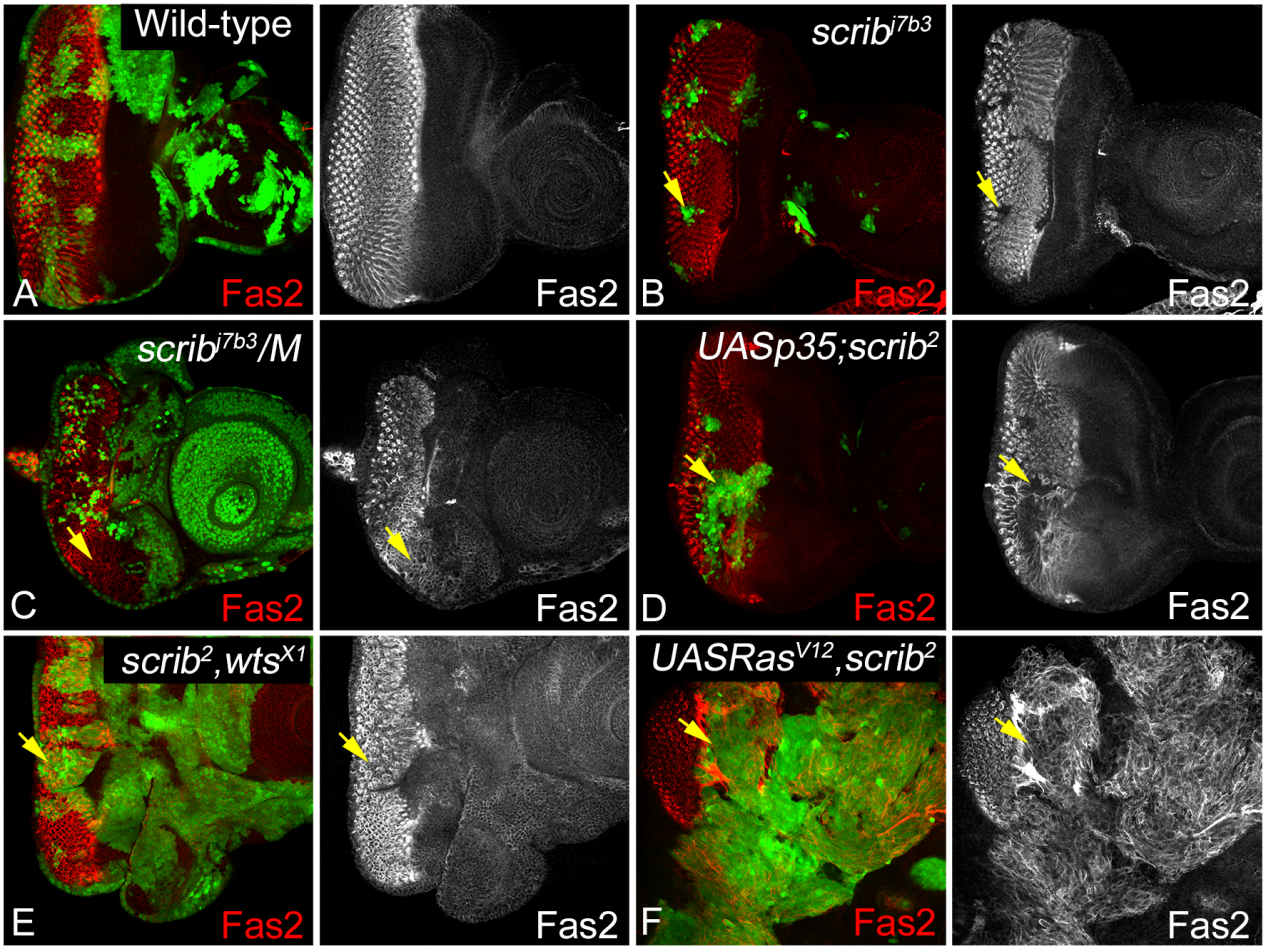
Alterations in Fas2 expression due to loss of *scrib*. Eye imaginal discs stained with antibody against *Drosophila* Fas2 (red, and greyscale in A-F) are shown in somatic clones of the genotypes (A) Wild-type (B) *scrib*^-^ (C) *scrib*^-^*/M*, (D) *p35+scrib*^-^, (E) *scrib*^-^,*wts*^-^, and (F) *Ras*^*V12*^,*scrib*^-^. The effect on Fas2 expression, and localization was assayed in clones that were present posterior to the MF where Fas2 is endogenously expressed. The yellow arrows in panels B-F point to the clones depicting changes in Fas2 expression.

Epithelial cells establish specific adhesion complexes at the lateral and apical cell surface that appear to act as specialized sites of signal transmission [63]. Fasciclin 2 (Fas2), the *Drosophila* Neural Cell Adhesion Molecule (NCAM) ortholog, is a member of the immunoglobulin superfamily that functions in lateral adhesion, growth cone guidance, and in reintegrating misoriented cells into epithelial monolayers [64–66]. In wild-type eye discs, Fas2 is strongly upregulated just posterior to the morphogenetic furrow, and is expressed basolaterally in the differentiated ommatidial clusters (Fig 3A). Interestingly, Fas2 expression is lost in *scrib*^-^ cells in wild type background (Fig 3B), and significantly reduced in *scrib*^-^*/M* (Fig 3C), *p35+scrib*^-^ (Fig 3D), and *Ras*^*V12*^,*scrib*^-^ (Fig 3F) clones respectively. However, in *scrib*^-^,*wts*^-^ (Fig 3E), *wts*^-^ (S3A Fig) or *UAS Ras*^*V12*^ (S3B Fig) clones, Fas2 expression is disrupted but not downregulated. Overall, these data suggest that loss of *scrib* is sufficient to downregulate Fas2 likely due to disruption of apical basal polarity and this effect is exaggerated in the different genetic backgrounds that modify the growth potential of *scrib* mutant cells. The loss of lateral adhesion generally makes cells susceptible to elimination from epithelial monolayers [64], however, the different growth potential of *scrib* mutant cells that co-express *p35* or *Ras*^*V12*^ suggests that other factors besides suppression of lateral adhesion or differentiation may contribute to the observed effects. Therefore, we tested if the apical adherence junctions are affected in *scrib* mutant cells in different genetic backgrounds.

**Fig 4:**
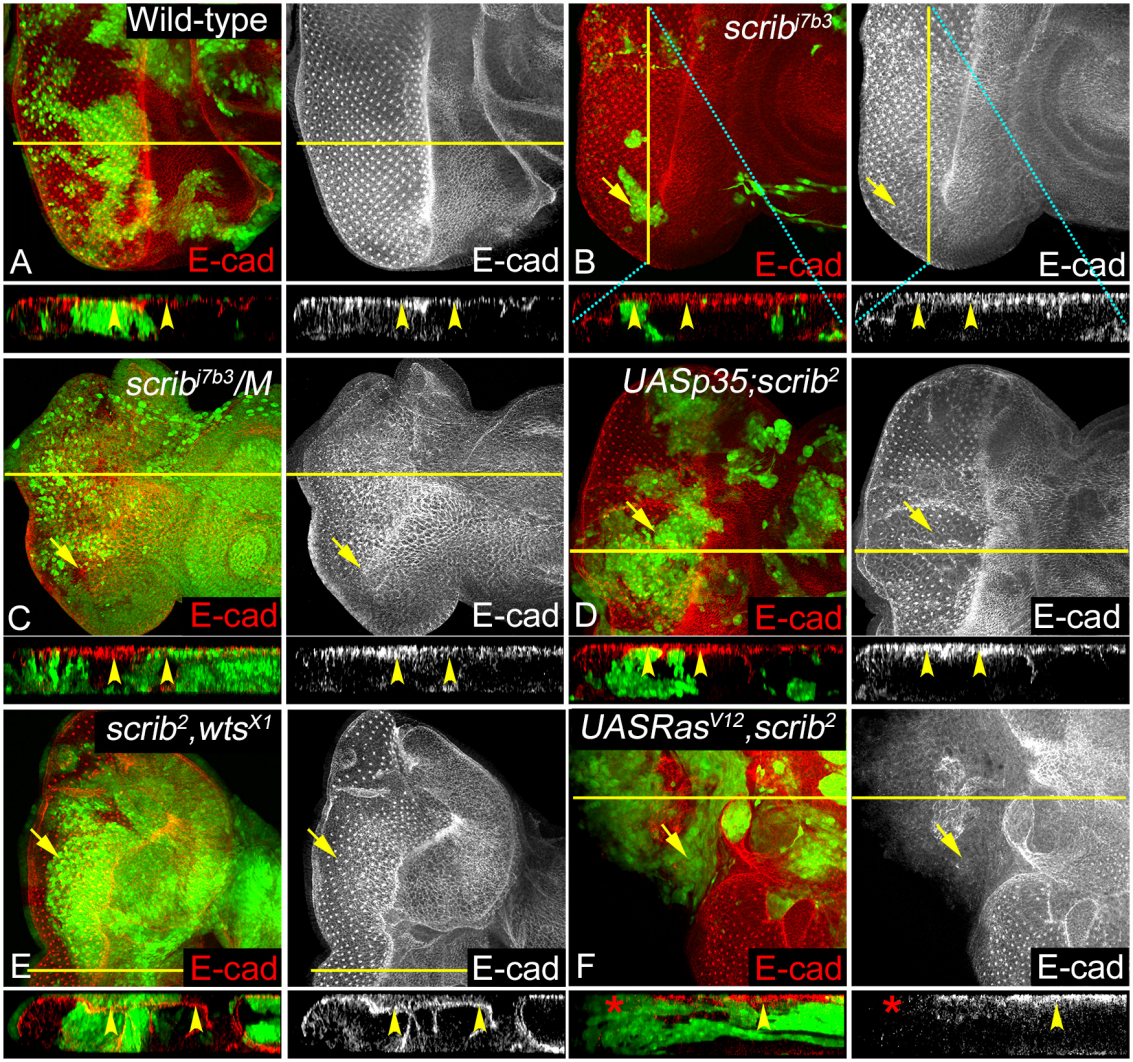
Changes in AJ organization in response to *scrib* loss of function in epithelial cells. Panels show eye imaginal discs stained with anti-E Cad antibody (red, and greyscale in A-F). Genotypes analyzed include (A) Wild-type (B) *scrib*^-^ (C) *scrib*^-^*/M*, (D) *p35+scrib*^-^, (E) *scrib*^-^,*wts*^-^, and (F) *Ras*^*V12*^,*scrib*^-^ clones. (A-F) Panels show expression of E-Cad staining in XY plane, clones marked with yellow arrows. In addition, XZ (A, C-F) or YZ (B, marked by cyan line) projections of region highlighted by yellow lines are shown for each genotype to show E-Cad localization. Yellow arrowheads in XZ or YZ projections (A-F) show apical E-Cad localization at the AJs, and red asterisk (F) shows lack of E-Cad in *Ras*^*V12*^, *scrib*^-^ clones.

E-Cad localizes to the AJs in epithelial cells, and is often downregulated or mislocalized in cancers [67–71]. In wild-type eye discs, E-Cad was localized normally at the AJs (Fig 4A). Interestingly, in *scrib*^-^ cells in wild-type background no obvious defect in E-Cad expression is seen (Fig 4B). Similarly, no appreciable change in E-Cad levels or localization (see Z-projections for all genotypes) is seen in *scrib*^-^*/M* (Fig 4C), *p35+scrib*^-^ (Fig 4D), *scrib*^-^,*wts*^-^ (Fig 4E), *wts*^-^ (S4A Fig), or *UAS Ras*^*V12*^ (S4B Fig) clones that show hyperplastic overgrowth. In contrast, in the *Ras*^*V12*^,*scrib*^-^ clones that show robust tumorigenic potential E-Cad is downregulated (Fig 4F) [30]. Overall, these data show that in all combinations except *Ras*^*V12*^,*scrib*^-^, E-Cad can localize correctly suggesting that although adhesion is reduced in the different genetic backgrounds that modify the growth potential of *scrib* mutant cells, the apical AJs may not be severely affected.

**Fig 5:**
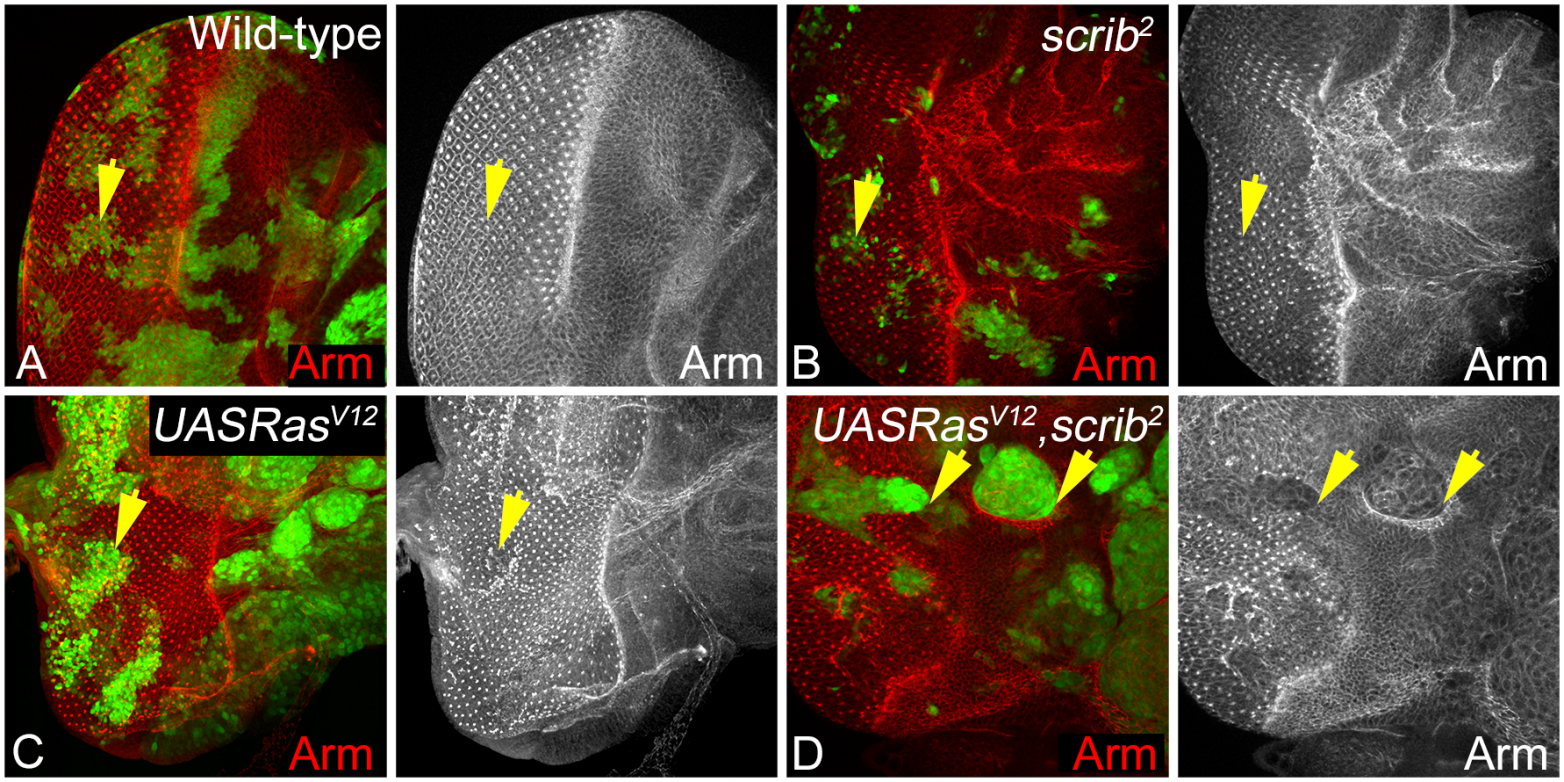
Armadillo expression is disrupted at the AJs during neoplastic growth. Arm expression and localization (red, and greyscale in A-D) in eye discs containing GFP positive *eyFLP* MARCM clones of the genotype (A) Wild-type, (B) *scrib*^-^, (C) *Ras*^*V12*^, and (D) *Ras*^*V12*^,*scrib*^-^ clones (GFP, green) is shown. The yellow arrows in panels A-D highlight mutant cells in different genotypes.

To further confirm our findings, we checked if levels and localization of another AJ protein- Armadillo (Arm) to test if AJs are affected in *scrib*^-^ or *Ras*^*V12*^,*scrib*^-^ mutant cells (Fig 5). In epithelial cells, Arm is ubiquitously expressed in the cytoplasm and the apical AJs, and anchors them to the actin cytoskeleton [23, 72]. Compared to wild-type (Fig 5A), expression of Arm is mildly disrupted in *scrib*^-^ clones especially posterior to the MF (Fig 5B). Similarly, in clones overexpressing *Ras*^*V12*^ alone Arm expression appears normal (Fig 5C). In contrast, in *Ras*^*V12*^,*scrib*^-^ clones, Arm levels are downregulated and mislocalized from the membrane to the cytoplasm (Fig 5D). These changes in Arm expression and localization are consistent with our earlier observations with E-Cad, confirming that loss of *scrib* alone does not significantly impact apical junctional complexes. In summary, epithelial integrity of *scrib* mutant cells is weakened due to disruption of the lateral but not apical cell adhesion in genetic backgrounds like *scrib*^-^*/M*, *p35+scrib*^-^, or *scrib*^-^,*wts*^-^ that enhance survival of *scrib*^-^ cells. However, in *Ras*^*V12*^,*scrib*^-^ clones both apical and translateral adhesion complexes are disrupted suggesting that cell adhesion is severely compromised. Overall, these data suggest that loss of differentiation is tightly correlated to loss of adhesion in *Ras*^*V12*^,*scrib*^-^ but not in other genetic backgrounds like *scrib*^-^*/M*, *p35+scrib*^-^, or *scrib*^-^,*wts*^-^ that enhance survival of *scrib*^-^ cells.

In addition to the reversible association of Arm with the AJs, cytoplasmic pools of Arm are regulated by phosphorylation-based mechanisms by Wingless (Wg) [73], and Jun N-terminal kinase (JNK) signaling pathway [74–76]. Interestingly, polarity regulators (like PAR-1) positively regulate Wnt/β-catenin pathway and inhibit the JNK pathway [77]. Thus, it is possible that downregulation of Arm in *Ras*^*V12*^,*scrib*^-^ clones is linked with increased JNK signaling previously reported in *Ras*^*V12*^,*scrib*^-^ clones. Further, increased JNK signaling is required for growth and invasion in *Ras*^*V12*^,*scrib*^-^ clones [30, 44]. Therefore, we tested levels of JNK signaling in all genetic combinations under study.

### JNK activation is not sufficient for Invasive growth of *scrib* mutant cells

**Fig 6:**
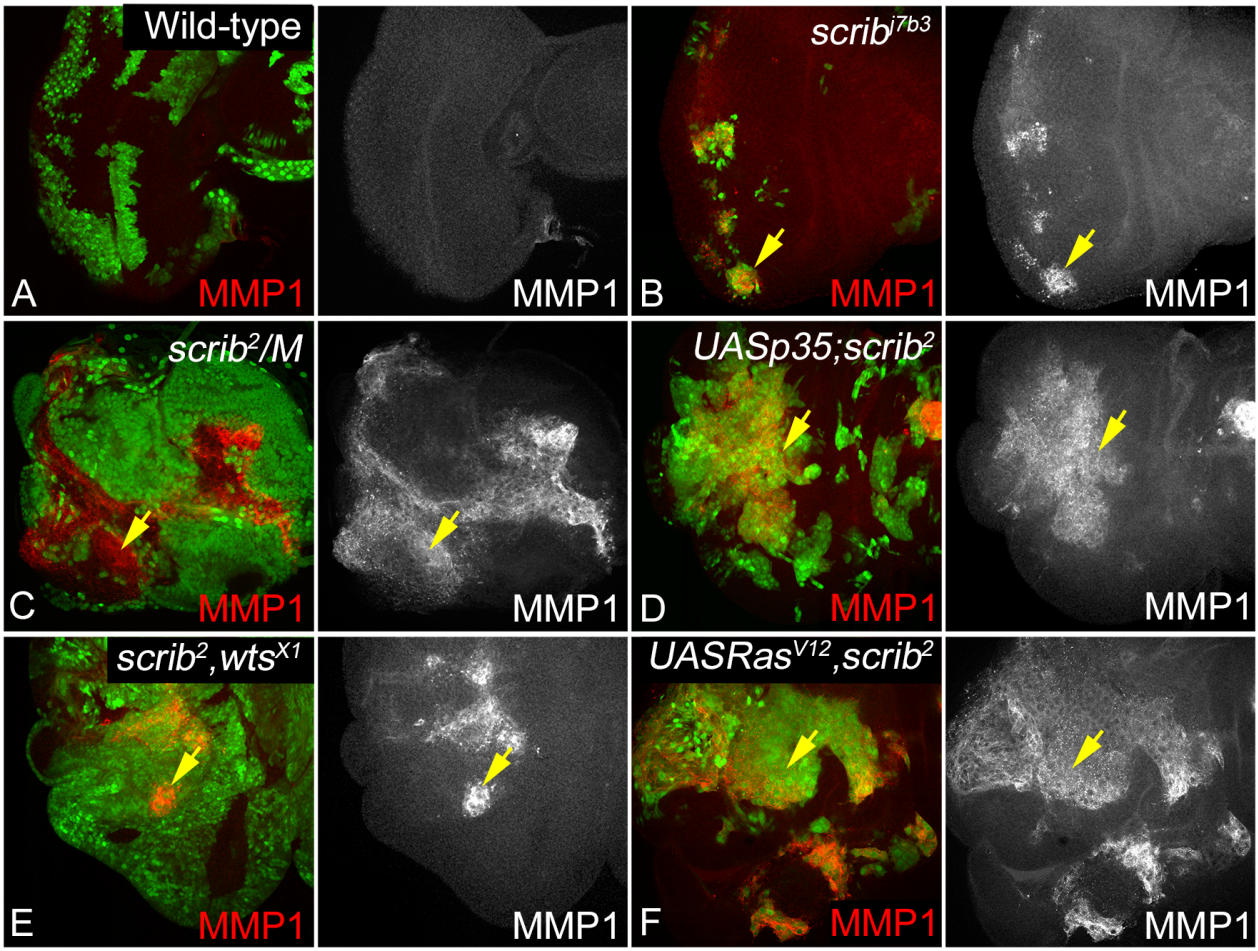
Effect of loss of *scrib* on JNK/MMP1 signaling. Panels show eye imaginal discs stained for MMP1 (red, greyscale in A-F) from the following genotypes: (A) Wild-type, (B) *scrib*^-^, (C) *scrib*^-^*/M*, (D) *p35+scrib*^-^, (E) *scrib*^-^,*wts*^-^, and (F) *Ras*^*V12*^,*scrib*^-^. Clones in A-B and D-F are GFP positive, and clones in C are GFP-negative, and are marked by yellow arrows.

Matrix metalloproteinase-1 (MMP1) is a JNK regulated gene that is a well-described marker of invasion, and is known to be involved in ECM degradation following EMT [78–81]. MMP1 also plays an important role in tissue remodeling and cell migration during development [80, 82]. Loss of *scrib* is known to induce JNK activity [30], however, do all genetic combinations under study show a similar induction with JNK activity, and if JNK activation was sufficient to confer invasive phenotype remains unclear. We therefore tested expression of MMP1 in the genotypes of our interest (Fig 6). MMP1 is expressed ubiquitously at low levels in wild type cells (Fig 6A). We observed a strong upregulation of MMP1 in all combinations under study, however, the pattern of MMP1 induction is variable. MMP1 is strongly induced in large clones in *scrib*^-^ (Fig 6B), *scrib*^-^*/M* (Fig 6C), *p35+scrib*^-^ (Fig 6D), and *Ras*^*V12*^,*scrib*^-^ (Fig 6F); however, MMP1 is induced in patches in *scrib*^-^,*wts*^-^ (Fig 6E) or *wts*^-^ (S5A Fig) clones. Interestingly, MMP1 is not induced in *UAS Ras*^*V12*^ control clones (S5B Fig). Taken together, these data suggest that loss of *scrib* is sufficient for JNK activation, however, JNK activation by itself does not correlate with tumorigenic growth. It is also interesting to note that MMP1 activation in all genetic combinations except *Ras*^*V12*^,*scrib*^-^ does not co-relate with invasion suggesting that invasive potential of *scrib* mutant cells is not just dependent on JNK activation but alteration of cell survival due to increased Epidermal Growth Factor/ Mitogen-Activated Protein Kinases (EGFR/MAPK) or Yki activity in case of *Ras*^*V12*^ or loss of *wts* respectively.

## Discussion

**Fig 7:**
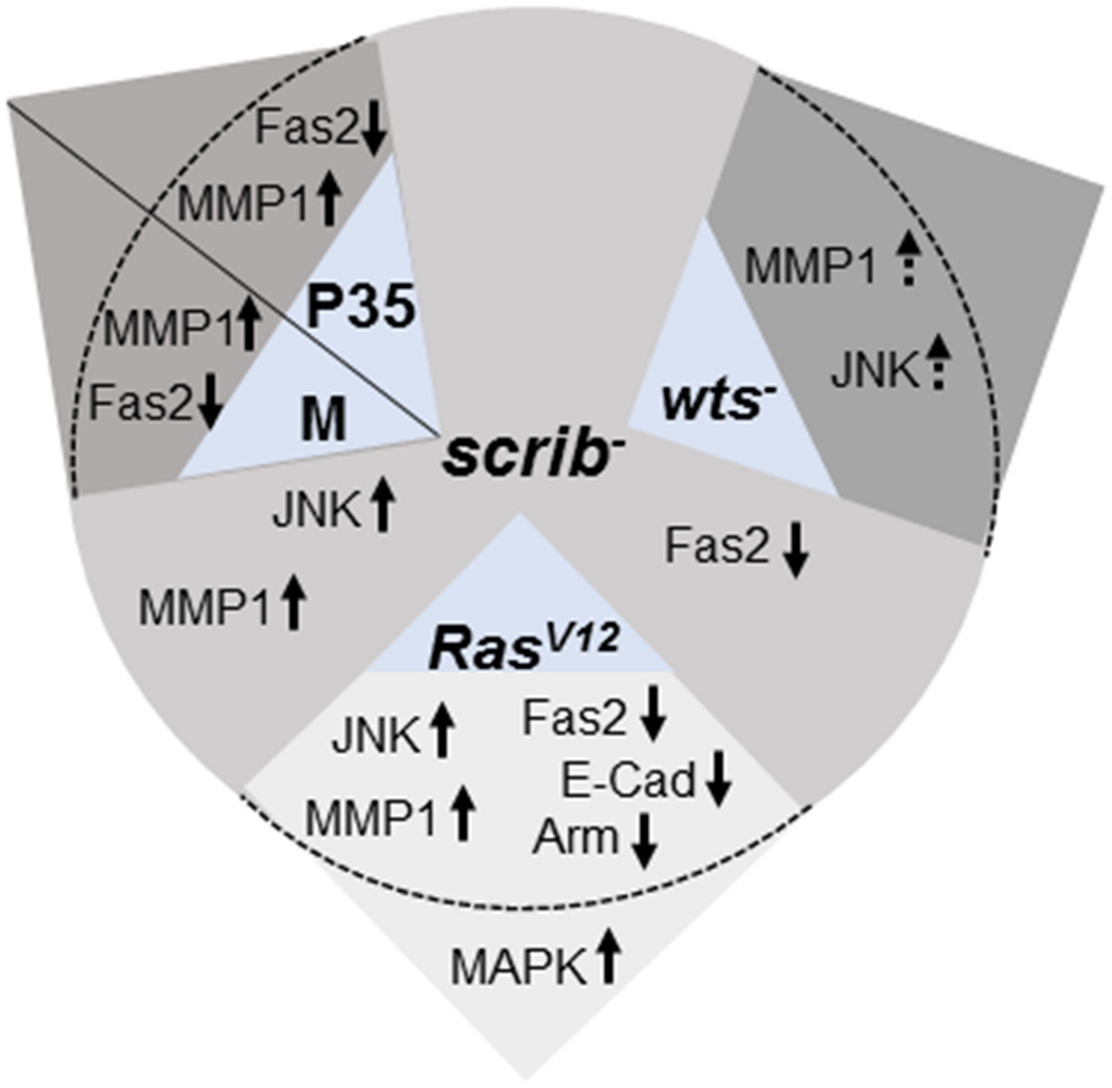
Models depicting changes in *scrib* mutant cells in different genetic backgrounds. The image shows changes in signaling pathways, and cell adhesion in *scrib* mutant cells, and changes to these signals in *scrib* cells combined with different growth modifiers.

Studies in *Drosophila* imaginal discs have provided important insights about effects of loss of apical basal polarity on cell proliferation, cell death and cooperative interactions that can lead to tumor growth and progression [1, 19, 20, 26, 27, 83]. In this study, we investigated if a central mechanism that regulates cell adhesion governs the growth and invasive potential of *scrib* mutant cells; and if this mechanism promotes tumorigenic growth via cooperative interactions. Over the last decade, a number of strategies have been used to study effects of loss of polarity by loss of function mutations in *scrib*, for example, loss of *scrib* in *Minute* background [28], or combining loss of *scrib* with *UASp35* which suppresses cell death [26, 84] or inducing *scrib* mutant clones in *eiger* mutant background [28, 84]. All of these manipulations lead to formation of *scrib* mutant cells that are no longer eliminated, and the range of phenotypes observed by loss of *scrib* in combination with these mutations is comparable but not identical, suggesting that these genetic modifiers of *scrib* induce distinct effects on growth and tumorigenesis. To address the shared and distinct effects of loss of *scrib* in different genetic backgrounds, we compared effects on proliferation, differentiation, cell survival and cell adhesion. Our studies show that reduced cell survival, activation of the JNK pathway and reduced cell adhesion are central to loss of *scrib*, however, for *scrib* cells to induce aggressive growth cooperative interactions that derail signaling pathways play an essential role in the mechanisms leading to tumorigenesis (Fig 7).

We initially compared differences in proliferation and differentiation, and their correlation to cell survival. In *Drosophila* eye imaginal discs, cell division is very tightly regulated in the differentiating cells posterior to the MF, where cells undergo a G1 arrest as they enter the MF, begin differentiating into the photoreceptor neurons to form the precluster (Fig 1). The cells then go through one round of cell division in the second mitotic wave and arrest in the G1 phase, and all the remaining cells that comprise the ommatidial clusters are differentiated [47]. We found that in *scrib* mutant clones generated in wild-type background, the cell division profile is largely unperturbed but in all other combinations mutant cells undergo ectopic proliferation and show defects in differentiation with respect to progression of the morphogenetic furrow or differentiation of photoreceptor neurons. Increased proliferation and loss of differentiation is linked to tumor progression, so we next tested if the genetic combinations that promote *scrib* growth affected differentiation (Fig 2). We found mild defects in morphogenetic furrow progression and spacing of ommatidial clusters in *scrib/M* cells, and *scrib*^-^ *wts*^-^ clones. We found that differentiation is suppressed in two genotypes (*p35+scrib*^-^, and *Ras*^*V12*^,*scrib*^-^) (Fig 2) where increased survival either by upregulation of DIAP1 or suppression of apoptosis by expression of *p35* promotes growth. However, *p35+scrib*^-^ clones do not grow into invasive tumors, suggesting that although loss of differentiation is linked to tumor progression, cells require a potent growth-promoting signal to form aggressive tumors. Furthermore, the differences in phenotype with respect to regulation of furrow progression and differentiation of photoreceptor neurons also shows that these genetic combinations do not share a single molecular mechanism to enhance growth of *scrib*^-^ cells.

Previous studies have shown that regulation of apical basal polarity and maintenance of junctional integrity plays a critical role in cellular functions and homeostasis [8, 25]. We tested if the genetic combinations that modify *scrib* growth and survival show any defects with respect to the lateral and apical adhesions formed by the homophilic binding of Fas2, E-Cad or Arm (Fig 7). Interestingly, Fas2 was misregulated in all genotypes, suggesting that loss of *scrib* resulted in disruption of photoreceptor differentiation and also defects in intercellular adhesion however, the severity of the phenotype is dependent on the modifying mutations. Furthermore, studies in other model systems have established an interesting reciprocal relationship between the regulation of NCAM (Fas2) and E-Cad in the initiation and maintenance of EMT [85]. Reduced levels of NCAM expression is shown to promote tumor dissemination *in vivo* [85]. NCAMs promote signaling changes in membrane microdomains, and promote interactions at the focal adhesion and AJs [9, 64]. Therefore, we extended our analysis to the stability of AJs (E-Cad, Arm) in the genetic combinations that promote growth of *scrib* mutant cells.

Consistent with previous data, where knockdown of *scrib* in wing discs caused no polarity defect [86], we found that loss of *scrib* in wild type or in *M* background did not result in loss of apical AJ proteins like E-Cad or Arm, suggesting that junctional organization is not immediately lost in these genetic combinations. Similarly, loss of *scrib* in combination with *wts* did not cause polarity defects. Comparison of AJ markers like E-Cad and Arm in *p35+scrib*^-^, and *Ras*^*V12*^,*scrib*^-^ sheds some light on the changes that may be critical for induction of invasive tumors. We found that both genotypes show loss of differentiation but adhesion is lost more severely in *Ras*^*V12*^,*scrib*^-^ mutant cells suggesting that apical basal polarity and cell adhesion are lost only in this genotype.

It is interesting to note that several key changes occur in *p35+scrib*^-^ cells, for example, defects in differentiation, loss of Fas2 and activation of MMP1, but these clones fail to induce robustly growing tumors. One reason for this may be that changes in cell adhesion in a cell where apoptotic signals are induced by activity of caspases, differ fundamentally from the changes that occur due to oncogenic cooperation. Normally, apoptotic cells show several key changes like condensation of the cytoplasm, breakdown of nuclear integrity, cell rounding, membrane blebbing, and in epithelial cells the loss of cell polarity and cell junctions [87, 88]. These changes are caused by cleavage of key proteins by caspases [89]. A key early change in the apoptotic cell is the loss of contact and extrusion of these cells from the epithelium by neighboring cells. Since the integrity of the epithelial cells depends on the cadherin-catenin mediated establishment of the apical AJs, it is not surprising that several of these junctional proteins are targets of caspase activity during apoptosis. For example, the *Drosophila* Caspase 3 Drice targets Armadillo for cleavage, which is responsible for the loss of *D*E-Cad from cell junctions and thus might contribute to the degeneration of epithelial integrity during apoptosis [90]. Our data with *p35+scrib*^-^ shows that expression of the caspase inhibitor prevents activation of caspases, thereby, causing no obvious change in the expression of E-Cad or Arm, which may be important for cells to change their signaling behavior and show robust tumorigenic growth. Taken together, our data show that loss of apical basal polarity and cell adhesion is critical for progression of *scrib* mutant cells to tumors. In addition, increased signaling from growth promoting pathways (Yki, TGF-β [Transforming growth factor beta], MAPK etc) synergistically contribute to tumor growth and progression.

We found that MMP1- a JNK regulated gene and marker for tumor invasion was induced in all genetic combinations under study (Figs 6 and 7). This was an interesting finding as *scrib/M* or *p35+scrib*^-^ or *scrib*^-^ *wts*^-^ clones show induction of MMP1, and an increase in clone size but do not show signature changes of aggressive neoplastic tumors. Thus, JNK activation is clearly not sufficient but required for the changes that confer tumorigenic potential to *scrib* mutant cells. It is thought that in *Ras*^*V12*^,*scrib*^-^ clones, JNK undergoes a paradoxical switch from pro-apoptotic to pro-proliferation signal by modifying Yki activity via inactivation of Wts by F-actin mediated activation of Ajuba [91]. However, direct inactivation of *wts* in *scrib* mutant cells shows a phenotype that is qualitatively different where clones show hyperplastic growth, and there is no loss of cell adhesion. Thus, oncogenic *Ras* specifically contributes to tumorigenesis by activation of MAPK and Yki that synergistically cause tumor growth and progression. Alternatively, AJs are disrupted only when certain clonal mass is achieved in *scrib*^-^ cells due to additional mutations such as *Ras*^*V12*^. In summary, our studies show that some changes are caused by loss of *scrib* and are therefore shared in all genotypes (e.g., effect on Fas2, MMP1) but other defects are contributed by the modifying mutations, which synergistically interact and modify the phenotype. In all genetic combinations where one or more of these critical changes do not occur show improved survival of *scrib* mutant cells, but not highly proliferative and invasive tumors. Overall our data show that loss of apical basal polarity and cell adhesion are critical for progression of *scrib* mutant cells to tumors. In addition, increased signaling from growth promoting pathways (Yki, TGFβ, MAPK, etc.) may synergistically contribute to tumor growth and progression.

## Supporting Information

S1 Fig Differentiation defects in *wts*^-^ and *Ras*^*V12*^ clones. Panels show ELAV (red, greyscale) expression in somatic clones (GFP, green) (A) *wts*^-^ loss of function, or (B) overexpression of *Ras*^*V12*^ in eye discs. ELAV staining within the clones is highlighted in yellow arrows.
(TIF)

S2 Fig Survival potential of *scrib*^-^ cells different genetic backgrounds. Panels show DIAP1 expression (Red, greyscale) in eye discs containing clones (GFP, green) of the following genotypes (A) wild-type, (B) *scrib*^-^, (C) *scrib*^-^/*M*, (D) *p35+scrib*^-^, (E) *scrib*^-^,*wts*^-^ and (F) *Ras*^*V12*^,*scrib*^-^ clones. Note that *scrib*^-^/*M* clones are marked by loss of GFP. DIAP1 expression in clones of the indicated genotypes is marked with yellow arrows.
(TIF)

S3 Fig Effect on Fas2 localization during hyperplastic growth. Eye imaginal discs showing Fas 2 expression (red, greyscale) in clones (GFP, green) of the genotype (A) *wts*^-^, and (B) *Ras*^*V12*^ are depicted. Note that clones located posterior to morphogenetic furrow (yellow arrows) are relevant for comparing changes in Fas2 expression.
(TIF)

S4 Fig E-Cad expression, and localization in *wts*^-^, and *Ras*^*V12*^ clones. E-Cad (red, greyscale) expression and localization in (A) *wts*^-^, and (B) *Ras*^*V12*^ clones (GFP, green) is shown. Panels show cross sections (of regions corresponding to yellow lines) to highlight E-Cad localization, and expression (yellow arrowheads). Cyan lines in A show the orientation of the YZ projection. Yellow arrows highlight E-Cad levels in appropriate clones.
(TIF)

S5 Fig MMP1 expression in *wts*^-^, and *Ras*^*V12*^ clones. Panels show eye discs containing *eyFLP* MARCM clones (marked by yellow arrows) (GFP, green) of the genotype (A) *wts*^-^, and (B) *Ras*^*V12*^ stained for MMP1 (red, greyscale).
(TIF)
