# Supplementary figures and images for: Loss of Cell Adhesion Increases Tumorigenic Potential of Polarity Deficient Scribble Mutant Cells
Source: PLoS One. 2016 Jun 21;11(6):e0158081. doi: 10.1371/journal.pone.0158081 (PMC4915667; doi:10.1371/journal.pone.0158081)

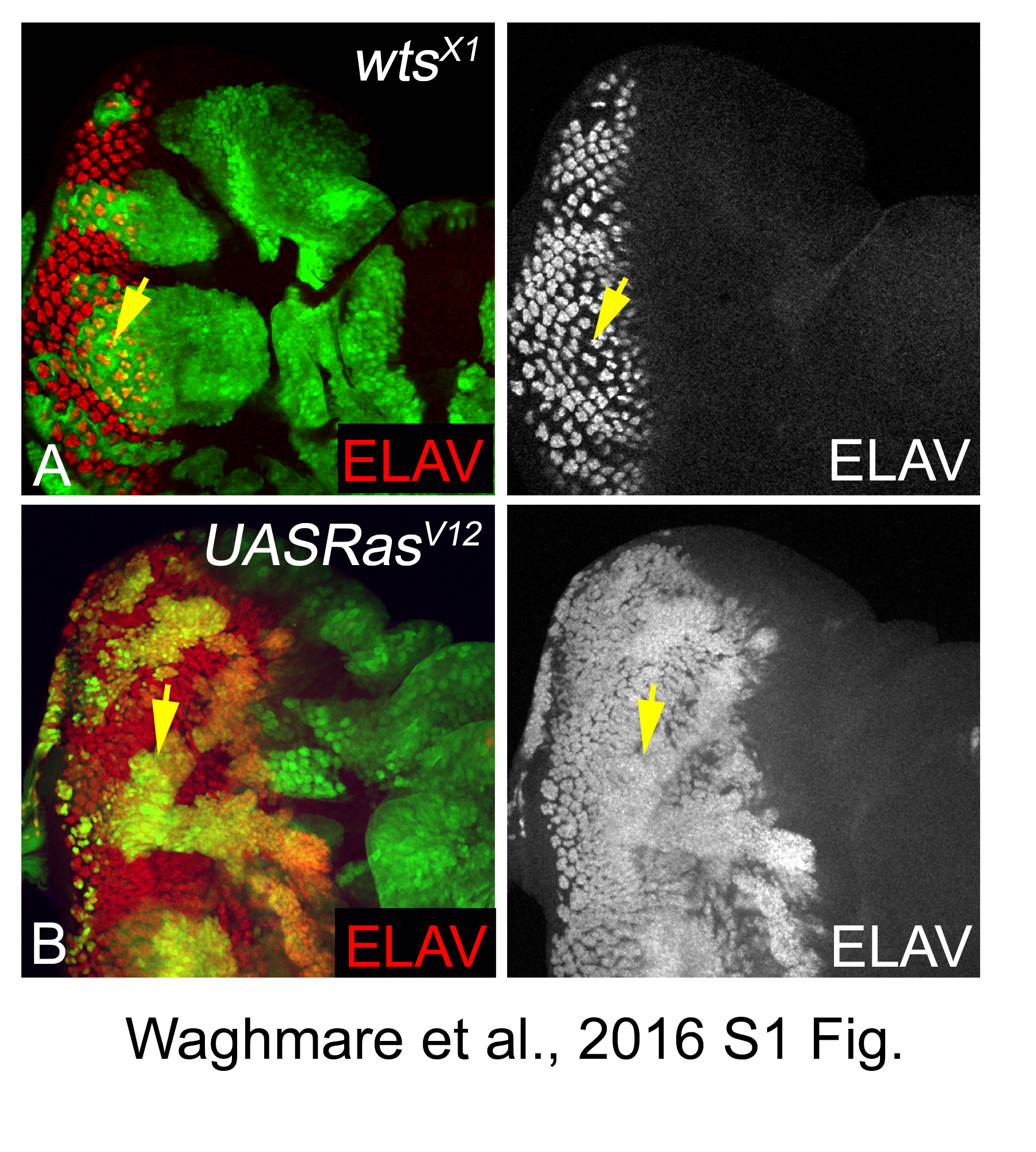

Supplement: S1 Fig — Panels show ELAV (red, greyscale) expression in somatic clones (GFP, green) (A) wts- loss of function, or (B) overexpression of RasV12 in eye discs. ELAV staining within the clones is highlighted in yellow arrows. (TIF) [file pone.0158081.s001.tif]

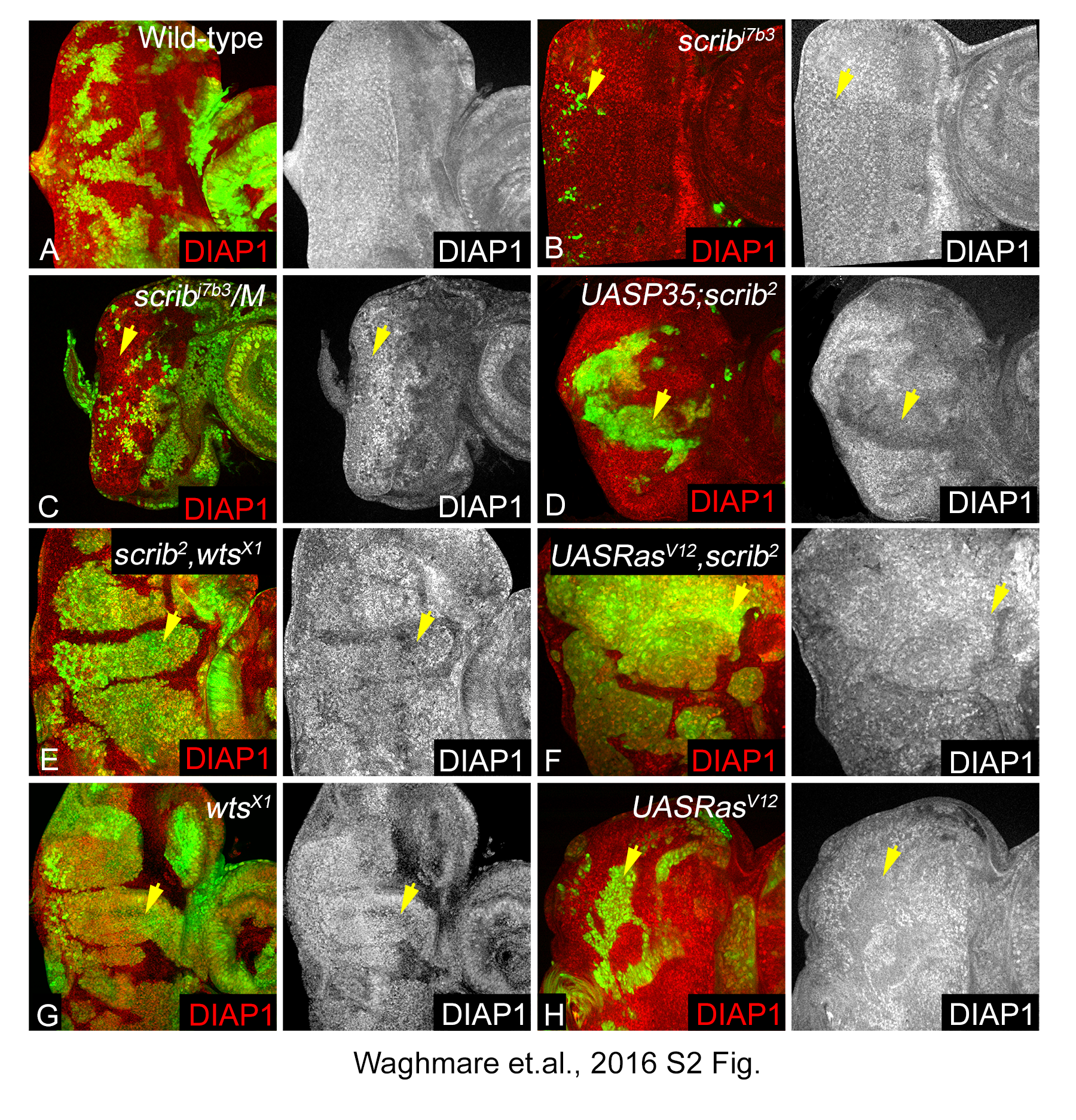

Supplement: S2 Fig — Panels show DIAP1 expression (Red, greyscale) in eye discs containing clones (GFP, green) of the following genotypes (A) wild-type, (B) scrib-, (C) scrib-/M, (D) p35+scrib-, (E) scrib-,wts- and (F) RasV12,scrib- clones. Note that scrib-/M clones are marked by loss of GFP. DIAP1 expression in clones of the indicated genotypes is marked with yellow arrows. (TIF) [file pone.0158081.s002.tif]

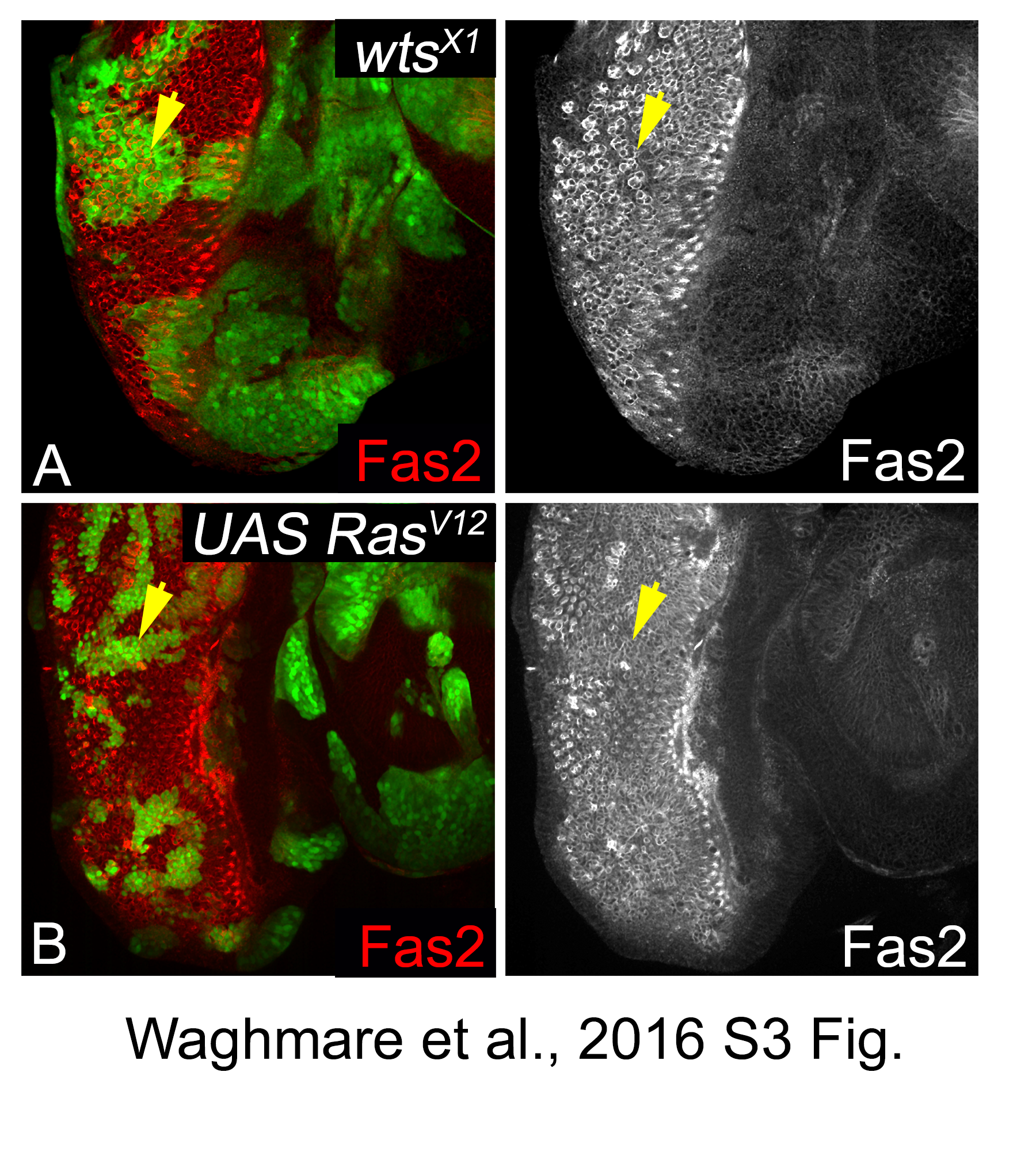

Supplement: S3 Fig — Eye imaginal discs showing Fas 2 expression (red, greyscale) in clones (GFP, green) of the genotype (A) wts-, and (B) RasV12 are depicted. Note that clones located posterior to morphogenetic furrow (yellow arrows) are relevant for comparing changes in Fas2 expression. (TIF) [file pone.0158081.s003.tif]

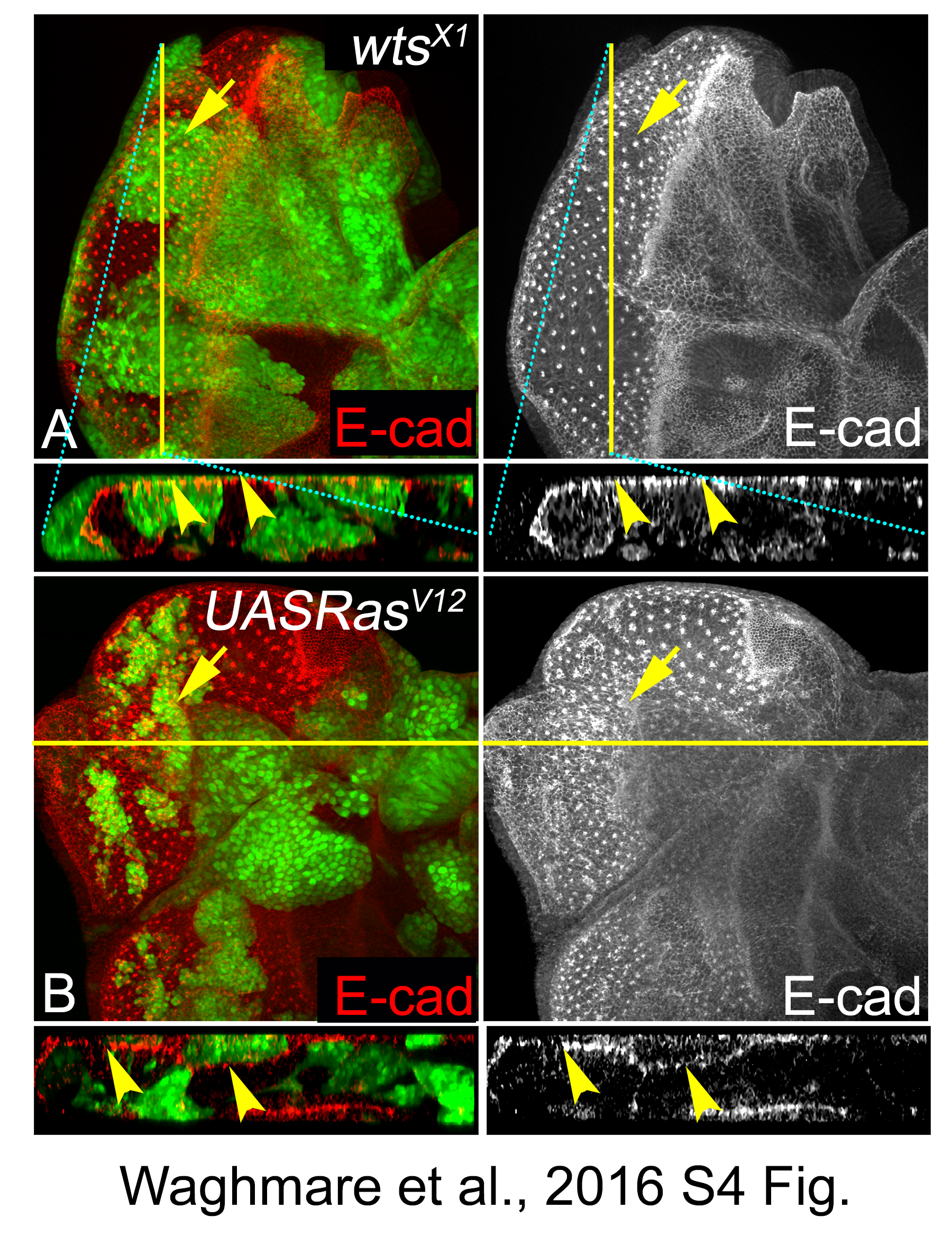

Supplement: S4 Fig — E-Cad (red, greyscale) expression and localization in (A) wts-, and (B) RasV12 clones (GFP, green) is shown. Panels show cross sections (of regions corresponding to yellow lines) to highlight E-Cad localization, and expression (yellow arrowheads). Cyan lines in A show the orientation of the YZ projection. Yellow arrows highlight E-Cad levels in appropriate clones. (TIF) [file pone.0158081.s004.tif]

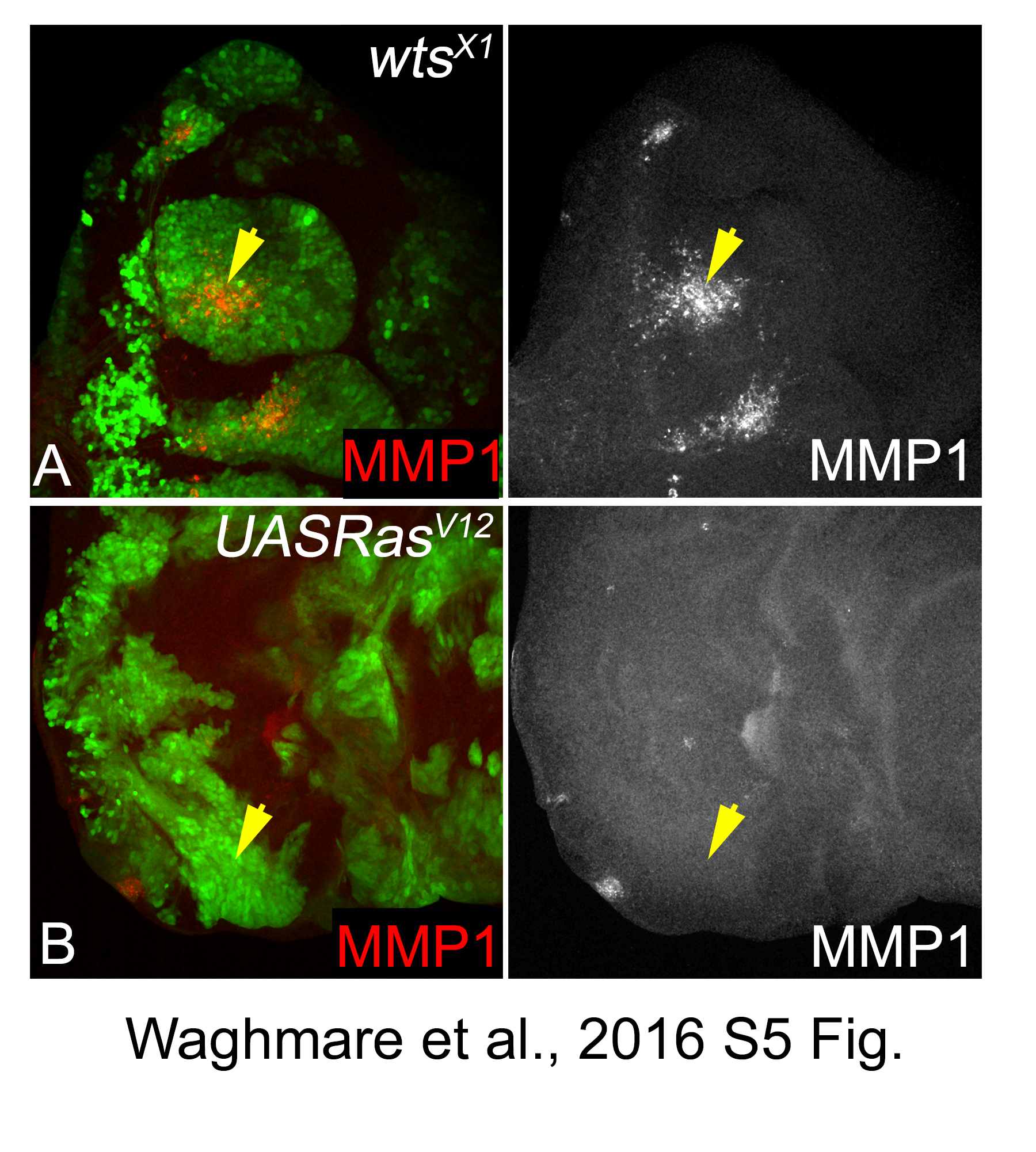

Supplement: S5 Fig — Panels show eye discs containing eyFLP MARCM clones (marked by yellow arrows) (GFP, green) of the genotype (A) wts-, and (B) RasV12 stained for MMP1 (red, greyscale). (TIF) [file pone.0158081.s005.tif]
